# The Physicians’ Pocket Day Book

**Published:** 1885-12

**Authors:** 


**The Physician's Pocket Day Book.** Designed by C. HENRI LEONARD, M. A., M. D., for 1886. Detroit: Illustrated Medical Journal Co.

There are a great many medical men who look upon this as the best book of the visiting lists. We have not ourselves used it, but it has advantages of its own which are possessed by no other. We would advise our readers to examine it before selecting their list for the ensuing year.

